# Exploring the prevalence and predictors of low resilience and likely PTSD in residents of two provinces in Canada during the 2023 wildfires

**DOI:** 10.3389/fpubh.2024.1343399

**Published:** 2024-03-25

**Authors:** Medard K. Adu, Reham Shalaby, Belinda Agyapong, Raquel da Luz Dias, Vincent I. O. Agyapong

**Affiliations:** ^1^Department of Psychiatry, Dalhousie University, Halifax, NS, Canada; ^2^Department of Psychiatry, University of Alberta, Edmonton, AB, Canada

**Keywords:** disaster, wildfires, post traumatic stress disorders, resilience, mental illness

## Abstract

**Background:**

The recent wildfires in Canada serve as a stark example of the substantial and enduring harm they cause to the health of individuals and communities. Assessing the prevalence and correlates of Post-traumatic stress disorder (PTSD) and low resilience is valuable for policymakers in public health.

**Objectives:**

The study aimed to assess the prevalence and predictors of low resilience and likely PTSD among subscribers of Text4Hope, an e-mental health program that delivered daily supportive messages to residents of Nova Scotia (NS) and Alberta(AB) during the recent wildfires.

**Method:**

Data collection was through a self-administered online survey completed by residents of the affected regions of NS and AB from May 14 to June 23, 2023. Data were analyzed using Statistical Package for the Social Sciences.

**Results:**

Out of 298 respondents, the prevalence of low resilience and likely PTSD in our sample were 52.0 and 39.3%, respectively. Unemployed respondents were about 3 times more likely to experience both low resilience and PTSD symptoms compared to those employed. Respondents with a history of mental health diagnosis were about 4 times more likely to experience likely PTSD compared to those with no history of mental health diagnosis.

**Conclusion:**

This study established that unemployment and a history of mental health diagnosis predicted likely PTSD, while unemployment was associated with low resilience during the wildfire. These findings offer insights for clinical interventions and the creation of psychosocial support programs for vulnerable populations.

## Introduction

Wildfire disasters have become a global environmental hazard that is experienced by communities worldwide. Recent wildfires in Canada demonstrate their significant and long-lasting impact on the health of individuals and the communities that are impacted, underscoring the importance of mitigation efforts (1). Wildfire is an essential ecological process that contributes to forest ecosystem health in Canada (2). According to Natural Resources Canada, reporting on the state of Canada’s forests in 2020 indicated that, wildfire disasters are among the most extensive disturbances in North American forests, and they affect over a million hectares annually (3–5).

Since the 1970s, trends suggest that the area burnt in Canada is on the rise, especially within the western regions (6) of which the changes are mostly due to the increasing severity of fire weather (7, 8). Wildfire disasters in Canada seem seasonal and usually occurs within the spring and summer months when there are drier and warmer weather condition favorable for fire combustion. The season of wildfires in Canada can vary from year to year and the severity of the impact of wildfires could largely be dependent upon the weather conditions, fuel availability, and human activities prevailing at a particular time. Geographically wildfires are predominant in various regions across Canada including but not limited to Alberta, Saskatchewan, British Columbia, Manitoba, Ontario, Quebec, and the Atlantic provinces (9). Wildfires have daring consequences on the environment, human health, wildlife, and infrastructure. There is often a loss of biodiversity, air pollution, destruction, and economic losses following wildfire disasters (4).

Wildfire disasters not only present with physical injuries and material losses but also go alongside emotional disturbances, psychosocial problems, and in the worst situations lasting mental health disorders which can last many decades post the adverse event (10). These psychological and mental health consequences are increasingly acknowledged; however, their assessment and management remain challenging. Data from a systematic review demonstrates that approximately 40% of individuals impacted by devastating natural disasters such as wildfires develop stress-related conditions like anxiety, PTSD, substance-induced/use disorders, and major depression (11, 12).

Findings from a scoping review conducted indicate a rise in the prevalence of PTSD in wildfire-affected communities, supported by statistical and clinical evidence (13). The criteria for diagnosing PTSD involve specific traumatic events, symptom combinations, and the absence of exclusionary factors (14). In the years following wildfires, adults experience elevated rates of post-traumatic stress disorder and its related symptoms, lasting for up to a decade (15, 16). A study involving 1,468 Greek adolescents after a wildfire revealed that 29.4% displayed probable PTSD symptoms 6 months later (14). Similarly, research on communities affected by Australian bushfires demonstrated that high-affected areas had more cases of PTSD compared to medium and low-affected areas, 3 to 4 years after the events (17).

This notwithstanding, certain studies that investigated mental health in community samples, found that only approximately 15% of adults who experienced life-threatening events developed long-term mental illnesses (18, 19). This suggests the importance of exploring other factors beyond the disaster itself as potential triggers to explain variations in individual responses.

The consensus in disaster-related literature is that mental health concerns should be an integral component of medical and emergency response efforts during and after post natural disaster (12). The World Health Organization’s 2018 panel of disaster management experts highlighted a crucial gap in addressing the long-term mental health consequences of disasters. They emphasized the need for continuous monitoring and the provision of specialized mental health care and psychosocial support to affected individuals and communities (20). Comprehensive awareness and analysis of risk factors for post-wildfire mental health problems benefit various stakeholders like clinicians, policymakers, and public health experts. This knowledge facilitates tailored prevention strategies to avert symptoms and aids in restoring patients’ recovery capacity (21).

According to the literature, an individual’s ability to function effectively after exposure to traumatic events signifies their successful adaptation and coping abilities (22, 23). The focus is on individual resilience post-trauma, defined by adversity and positive adaptation (24). Resilience involves maintaining a healthy lifestyle after stress, fostering personal growth and recovery (24). Low resilience is a concern in the general population and disaster victims, potentially rising due to natural disasters. This is of particular concern because of the significance of an individual’s capacity to cope with both the physical and psychological challenges during and following a severe disaster. This ability plays a crucial role in shaping the long-term mental health outcomes for those affected by such disasters (25). For instance, PTSD prevalence among directly impacted victims ranges from 30 to 40% (11). The differences in the prevalence of longer-term mental health impacts post-disasters can be attributed to various factors. Sociodemographic and clinical predictors have been identified, including individual resilience, disaster severity, coping abilities, victim involvement, age, and sex (26). For instance, women often experience psychological effects due to their domestic responsibilities, while children are more vulnerable and less resilient to natural disasters compared to adults (27–29).

Recently in Canada, a series of unprecedented wildfires has been ongoing since March 2023, with a notable intensification in June (30). This wildfire season is believed to have broken records, making it the most severe in the country’s history. The wildfires are expected to affect the largest area ever recorded. These fires have impacted eleven provinces and territories, including Alberta, Nova Scotia, Ontario, and Quebec, causing significant damage to the natural environment. The effects of these wildfires are not limited to the fire-affected regions; they also extend to cities hundreds of miles away, such as Toronto and New York. These urban areas are experiencing and closely observing the consequences (30). As part of measures to provide support for individuals needing mental health support in the affected regions of Alberta and Nova Scotia during the peak of the recent wildfire outbreak, the Global Psychological E-health Foundation launched its renowned supportive text message intervention (Text4Hope) to lend mental health support to those needing it. As part of the intervention rendered to the affected population, we conducted this study to evaluate the extent of the impact of the wildfires on the mental health of survivors. Therefore, this study aimed to explore the occurrence and predictors of low resilience and likely PTSD among respondents and analyzed various potential factors, including demographics, clinical variables, and other risks, using self-administered surveys through RedCap.

Adequate knowledge and understanding of the risk factors for the development of symptoms of low resilience and PTSD after wildfire disasters can help stakeholders provide suitable preventative measures for the affected individuals to minimize long-term mental health problems and enable individuals to build resilience to aid early recovery. This understanding could potentially aid countries that like Canada, are prone to natural disasters, in effectively supporting individuals vulnerable to post-disaster mental health issues in the future.

## Methods

### Study design, consent, and institutional review board approval

This was a cross-sectional study. Data was collected through a self-administered online survey completed by residents of Alberta and Nova Scotia. The survey was administered via Redcap, a secure browser-based application for building and managing online surveys and translational research databases (31). Respondents were included if they were aged 18 years and above, residents of Alberta and Nova Scotia at the time of the wildfire and its evacuation processes, and they received the online-based self-administered questionnaire.

A total sample of 1802 surveys were received from the two provinces and the exclusion of incomplete responses yielded 298 complete surveys. The questionnaire was distributed randomly via email using government, school, occupational, and community platforms. Consent was implied by completing the survey. The study was conducted according to the guidelines of the Declaration of Helsinki and received ethical approval from the Health Research Ethics Board of the University of Alberta (Pro00086163) and the Research Ethics Board at Nova Scotia Health (REB file #1028254).

### Sample size estimation

To estimate the prevalence of low resilience and likely PTSD, considering a population of 5,232,018 made up of 969,383 and 4,262,635 from Nova Scotia and Alberta, respectively, based on the 2021 census, a 95% confidence interval, and a margin of error of ±5%, a sample size of 385 individuals was determined as necessary for this study.

### Data collection

Data collection occurred between May 14 and June 23, 2023, with a survey taking 5–10 min to complete. The survey covered sociodemographic factors (e.g., gender, age, ethnicity, marital status, employment status, educational status, and housing status) and mental health history (depression, anxiety, psychotropic medications).

Wildfire-related variables were also collected and included living in a region of Alberta or Nova Scotia that has recently been impacted by the wildfires and the frequency of watching television images about the devastation caused by the wildfires in the two provinces. The survey further accessed questions concerning the evacuation status, the losses attributed to the wildfires, and the support received during this devastating event.

The survey measured PTSD symptoms using the PTSD Checklist Civilian (PCL-C) (32, 33), a self-report scale that measures PTSD presence and severity. The 17-item checklist corresponds to the PTSD symptoms as stated by the Diagnostic and Statistical Manual (DSM-IV). The level of distress produced by each symptom is rated from 1 (not at all) to 5 (extremely). A score > 44 is deemed clinically significant (maximum score = 85). The PCL-C has been shown to have good reliability and convergent validity (34). The PCL-C has been shown to have good reliability and convergent validity (32). On the other hand, the Brief Resilience Scale (BRS) (35) was used to assess resilience as the main outcome variable of the study. BRS is made up of six questions. Items 1, 3, and 5 are positively worded, and items 2, 4, and 6 are negatively worded. For analysis, we compiled normal and high resilience into one category to compare to low resilience. The BRS is scored by reverse coding items 2, 4, and 6 and finding the mean of the six items. The following instructions are used to administer the scale: “Please indicate the extent to which you agree with each of the following statements by using the following scale: 1 = strongly disagree, 2 = disagree, 3 = neutral, 4 = agree, 5 = strongly agree.”

These scales have been validated and used in similar research previously conducted in Canada (36).

### Data analysis

Data were analyzed using Statistical Package for the Social Sciences (SPSS) Version 25 (IBM Corp 2011) (15). Demographic characteristics, wildfire-related variables dependent on the province of affiliation, and feedback to questions related to resilience and PTSD were summarized by absolute numbers and percentages. Chi-squared analysis was performed to assess the association of diverse study variables and the two outcome variables individually, including the resilience categorical variables (low and high-to-normal resilience) and the PTSD categorical variables (unlikely PTSD and Likely PTSD). Logistic regression analysis was performed to identify independent significant predictors of low resilience and likely PTSD variable conditions in two different models. From the Chi-square analysis, variables which had significant (*p* ≤ 0.05) or near significant (0.1 > *p* > 0.05) association with low resilience and likely PTSD were included in their respective regression model. A diagnostic correlation analysis was performed prior to regression analysis to exclude the high inter-correlation between predictor variables. Odds ratios (OR) and confidence intervals from the binary logistic regression analysis were calculated to determine the association between the predictor variables and the presence of low resilience and likely PTSD controlling for the other variables in each model.

## Results

**Figure 1:**
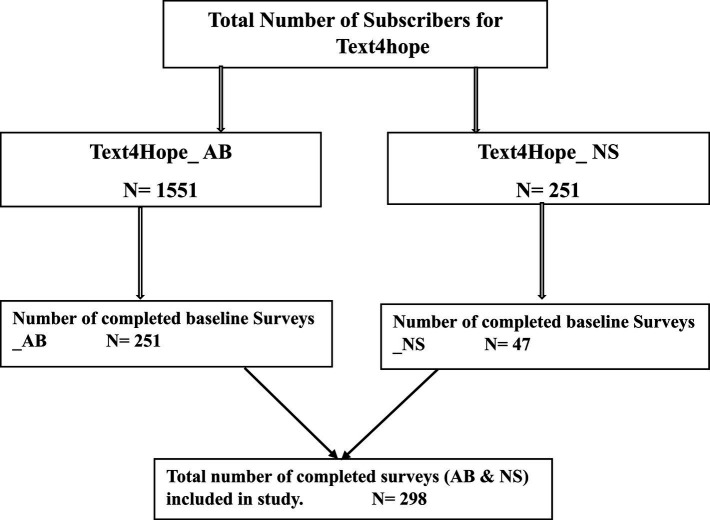
Study flow chart.

Figure 1 shows the total number of subscribers from May 14 to June 23, 2023, in Alberta (1551), with a total of 251 providing complete responses at baseline (response rate = 16.9%).

A total of 251 subscribed to the service from Nova Scotia with 47 responders providing complete responses at baseline (response rate = 18.7%).

As displayed in Table 1, the median age for respondents was 46 and 50 for respondents from NS and AB, respectively. Most respondents 84 (28.3%) were ≤ 40 years of age, 253 (85.2%) were females, 248 (83.5%) belonged to the Caucasian ethnic group, 246 (82.8%) had post-secondary education, 167 (56.4%) were in a relationship, 189 (63.6%) were employed, 200 (67.3%) lived in their own homes at the time of this study.

**Table 1:** Demographic profile, clinical characteristics, and wildfire-related items across the two provinces.

Variables	Nova Scotia *n* (%)	Alberta *n* (%)	Total *n* (%)
**Age**			
Median	46.00	50.00	
Mean (SD)	47.11 (1.92)	48.62 (0.89)	
**Age categories**			
≥ 60y	7 (14.9)	65 (26.0)	72 (24.2)
50–59	15 (31.9)	65 (26.0)	80 (26.9)
40–49	10 (21.3)	51 (20.4)	61 (20.5)
≤40y	15 (31.9)	69 (27.6)	84 (28.3)
**Gender**			
Male	7 (14.9)	31 (12.4)	38 (12.8)
Female	38 (80.9)	215 (86.0)	253 (85.2)
Other	2 (4.3)	4 (1.6)	6 (2.0)
**Ethnicity**			
Caucasian	39 (83.0)	209 (83.6)	248 (83.5)
Indigenous	0 (0.0)	18 (7.2)	18 (6.1)
Asian	0 (0.0)	11 (4.4)	11 (3.7)
Black/Hispanic	5 (10.6)	4 (1.6)	9 (3.0)
Other	3 (6.4)	8 (3.2)	11 (3.7)
**Education level**			
High School or Lower Education	7 (14.9)	44 (17.6)	51 (17.2)
Post-secondary Education	40 (85.1)	206 (82.4)	246 (82.8)
**Relationship status**			
In a relationship	32 (68.1)	135 (54.2)	167 (56.4)
Not in a relationship	15 (31.9)	114 (54.8)	129 (43.6)
**Employment status**			
Employed	32 (68.1)	157 (62.8)	189 (63.6)
Unemployed	15 (31.9)	93 (37.2)	108 (36.4)
**Housing status**			
Own home	33 (70.2)	167 (66.8)	200 (67.3)
Renting accommodation	11 (23.4)	53 (21.2)	64 (21.5)
Live with family or friend	3 (6.4)	30 (12.0)	33 (11.1)
**History of mental health diagnosis**			
Depression			
No	29 (61.7)	101 (40.2)	130 (43.6)
Yes	18 (38.3)	150 (59.8)	168 (56.4)
Bipolar Disorder			
No	43 (91.5)	239 (95.2)	282 (94.6)
Yes	4 (8.5)	12 (4.8)	16 (5.4)
Anxiety			
No	24 (51.1)	117 (46.6)	141 (47.3)
Yes	23 (48.9)	134 (53.4)	157 (52.7)
Alcohol abuse			
No	43 (91.5)	243 (96.8)	286 (96.0)
Yes	4 (8.5)	8 (3.2)	12 (4.0)
Drug abuse			
No	45 (95.7)	242 (96.4)	287 (96.3)
Yes	2 (4.3)	9 (3.6)	11 (3.7)
Schizophrenia			
No	46 (97.9)	249 (99.2)	295 (99.0)
Yes	1 (2.1)	2 (0.8)	3 (1.0)
Personality Disorder			
No	42 (89.4)	236 (94.0)	278 (93.3)
Yes	5 (10.6)	15 (6.0)	20 (6.7)
PTSD			
No	42 (89.4)	237 (94.4)	279 (93.6)
Yes	5 (10.6)	14 (5.6)	19 (6.4)
ADHD			
No	46 (97.9)	238 (94.8)	248 (95.3)
Yes	1 (2.1)	13 (5.2)	14 (4.7)
No mental health diagnosis			
No	29 (61.7)	206 (82.1)	235 (78.9)
Yes	18 (38.3)	45 (17.9)	63 (21.1)
Other			
No	46 (97.9)	248 (98.8)	294 (98.7)
Yes	1 (2.1)	3 (1.2)	4 (1.3)
History of psychotropic medications*			
Antidepressants			
No	30 (63.8)	152 (60.6)	182 (61.1)
**Yes**	17 (36.2)	99 (39.4)	116 (38.9)
Antipsychotics			
No	42 (89.4)	235 (93.6)	277 (93.0)
Yes	5 (10.6)	16 (6.4)	21 (7.0)
Benzodiazepines			
No	42 (89.4)	240 (95.6)	282 (94.6)
Yes	5 (10.6)	11 (4.4)	16 (5.4)
Mood stabilizers			
No	43 (91.5)	228 (90.8)	271 (90.9)
Yes	4 (8.5)	23 (9.2)	27 (9.1)
Sleeping tablets			
No	44 (93.6)	221 (88.0)	265 (88.9)
Yes	3 (6.4)	30 (12.0)	33 (11.1)
Stimulants for ADHD			
No	46 (97.9)	242 (96.4)	288 (96.6)
Yes	1 (2.1)	9 (3.6)	10 (3.4)
Other			
No	46 (97.9)	242 (96.4)	288 (96.6)
Yes	1 (2.1)	9 (3.6)	10 (3.4)
Not on any psychotropic medication			
No	22 (46.8)	128 (51.0)	150 (50.3)
Yes	25 (53.2)	123 (49.0)	148 (49.7)
**Having received MH counselling in the past year**
No	25 (53.2)	121 (48.4)	146 (49.2)
Yes	22 (46.8)	129 (51.6)	151 (50.8)
**Living in a region of AB/NS that has recently been impacted by the wildfires**
No	19 (40.4)	166 (66.1)	185 (62.1)
Yes	28 (59.6)	85 (33.9)	113 (37.9)
**Having a wildfire evacuation order issued for the subscriber area of residence**
Yes	9 (32.1)	19 (22,4)	28 (24.8)
No	19 (67.9)	59 (69.4)	78 (69.0)
Not applicable	0 (0.0)	7 (8.2)	7 (6.2)
**Having had to evacuate from your home due to the recent wildfires in AB/NS**
No	20 (71.4)	68 (80.0)	88 (77.9)
Yes	8 (28.6)	17 (20.0)	25 (22.1)
**Have you lost any property because of the wildfire?**
No	27 (96.4)	82 (96.5)	109 (96.5)
Yes	1 (3.6)	3 (3.5)	4 (3.5)
**Kind of property that was lost**			
Home	1 (2.1)	2 (0.8)	3 (1.0)
Car	1 (2.1)	1 (0.4)	2 (0.7)
Farm	0 (0.0)	0 (0.0)	0 (0.0)
**Having received support from family and friends in relation to the recent wildfire**
Absolute support	11 (39.3)	17 (20.0)	28 (24.8)
Some support	7 (25.0)	17 (20.0)	24 (21.2)
Only limited support	3 (10.7)	15 (17.6)	18 (15.9)
Not at all	7 (25.3)	36 (42.4)	43 (38.1)
**Having received support from the government of AB/NS in relation to the recent wildfire**
Absolute support	1 (3.6)	5 (5.9)	6 (5.3)
Some support	5 (17.9)	8 (9.4)	13 (11.5)
Only limited support	3 (10.7)	5 (5.9)	8 (7.1)
Not at all	19 (67.9)	67 (78.8)	86 (76.1)
**Having received support from the Red Cross in relation to the recent wildfire**
Some support	4 (14.3)	2 (2.4)	6 (5.4)
Only limited support	1 (3.6)	3 (3.6)	4 (3.6)
Not at all	23 (82.1)	79 (94.0)	102 (91.1)
**Frequency of watching television images about the devastation caused by the recent wildfires in AB/NS**
Daily	34 (72.3)	71 (28.4)	105 (35.4)
About every other day	7 (14.9)	52 (20.8)	59 (19.9)
About once a week	1 (2.1)	30 (12.0)	31 (10.4)
Less than once a week	2 (4.3)	36 (14.4)	38 (12.8)
Haven’t watched TV images of the devastation	3 (6.4)	61 (24.4)	64 (21.5)
**Having called the Mental Health Criss line in relation to the recent wildfires in AB/NS**
No	42 (89.4)	251 (100.0)	293 (98.3)
Yes	5 (10.6)	0 (0.0)	5 (1.7)
**Resilience**			
High-to-normal resilience	25 (56.8)	98 (46.2)	123 (48.0)
Low resilience	19 (43.2)	114 (53.8)	133 (52.0)
**PTSD**			
Unlikely PTSD	26 (61.9)	122 (60.4)	148 (60.7)
Likely PTSD	16 (38.1)	80 (39.6)	96 (39.3)

Regarding clinical variables, 168 (56.4%) had a history of mental health diagnosis for MDD, 157 (52.7%) for anxiety, 16 (5.4%) received mental health diagnosis of bipolar disorder, 12 (4.0%) for Alcohol Abuse, 20 (6.7%) for personality disorder, 19 (6.4%) for PTSD, and 63 (21.1%) reported they have no prior mental health diagnoses. Regarding medication, respondents who reported that they received antidepressants were 116 (38.9%), 21 (7.0%) were on antipsychotics, 16 (5.4%) were on Benzodiazepines, 27 (9.1%) were on mood stabilizers, 33 (11.1%) were on sleeping tablets, 10 (3.4%) were on other medications, and 148 (49.7%) reported they were not on any psychotropic medications.

Majority of the respondents 151 (50.8%), answered “no” to receiving mental health counselling in the past year, 185 (62.1%) responded living in a region of AB/NS that has recently been impacted by the wildfires, 28 (24.8%) had a wildfire evacuation order issued for their area of residence, 25 (22.1%) responded yes to having had to evacuate from their home due to the recent wildfires in AB/NS. The majority of respondents who lived in the wildfire impacted region reported not receiving any wildfire related support from either family and friends 43 (38.1%), the government of AB/NS 86 (76.1%), or from Red Cross 102 (91.1%). The majority 105 (35.4%), otherwise, responded to watching daily television images about the devastation caused by the wildfires in AB/NS. 293 (98.3%) denied they called the Mental Health Criss line concerning the recent wildfires in AB/NS, while 133 (52.0%) and 96 (39.3%) met the criteria of Low resilience and likely PTSD on BRS and PCL-C scales, respectively.

A summary of the results of the univariate analysis of the association between the study variables and low resilience and the likelihood of PTSD is displayed in Table 2. Fourteen variables showed a significant relationship with low resilience symptoms, and 17 variables showed a significant relationship with likely PTSD.

**Table 2:** Association analysis of demographic, clinical, and wildfire characteristics against low resilience and likely PTSD parameters.

Variables	Low resilience			Likely PTSD		
	*N* (%)	Chi-square*	*p*-value	*N* (%)	Chi-square*	*p*-value
**Province**						
NS	19 (43.2)	1.64	0.20	16 (38.1)	0.03	0.86
AB	114 (53.8)			80 (39.6)		
**Age categories**						
≥ 60y	26 (41.9)	11.28	0.01	18 (30.0)	13.70	0.00
50–59	33 (50.8)			26 (42.6)		
40–49	24 (43.6)			14 (25.9)		
≤40y	50 (67.6)			38 (55.1)		
**Gender**						
Male	11 (36.7)			11 (40.7)		
Female	118 (53.6)	0.16*	0.17	84 (39.8)	0.61	0.51
Other	4 (66.7)			1 (16.7)		
**Ethnicity**						
Caucasian	109 (50.5)			75 (36.2)		
Indigenous	11 (68.8)			8 (53.3)		
Asian	5 (50.0)	0.52*	0.51	4 (40.0)	0.06	0.07
Black/Hispanic	3 (42.9)			4 (66.7)		
Other	5 (71.4)			5 (83.3)		
**Education level**						
High School or Lower Education	22 (59.5)	0.98	0.32	17 (51.5)	2.37	
Post-secondary Education	111 (50.7)			79 (37.4)		0.12
**Relationship status**						
In a relationship	70 (47.6)	2.60	0.11	46 (32.6)	6.32	0.01
Not in a relationship	63 (57.8)			50 (48.5)		
**Employment status**						
Employed	76 (45.2)	8.83	0.00	52 (32.1)	10.60	0.00
Unemployed	57 (64.8)			44 (53.7)		
**Housing status**						
Own home	83 (48.5)			52 (32.1)		
Renting accommodation	34 (57.6)	2.52	0.28	30 (51.7)	10.92	0.00
Live with family or friend	16 (61.5)			14 (58.3)		
**History of mental health diagnosis**						
Depression						
No	38 (35.8)	18.80	0.00	22 (22.0)	21.36	0.00
Yes	95 (63.3)			74 (51.4)		
Bipolar Disorder						
No	125 (51.7)	0.16	0.69	88 (37.9)	3.95	0.05
Yes	8 (57.1)			8 (66.7)		
Anxiety						
No	37 (33.3)	27.22	0.00	28 (26.2)	13.86	0.00
Yes	96 (66.2)			68 (49.6)		
Alcohol Abuse						
No	126 (51.6)	0.21	0.65	89 (38.2)	2.85	0.09
Yes	7 (58.3)			7 (63.6)		
Drug Abuse						
No	128 (52.2)	0.19	0.66	89 (38.0)	4.11	0.04
Yes	5 (45.5)			7 (70.0)		
Schizophrenia						
No	132 (52.2)	0.61*	0.52	96 (39.7)	0.52	0.25
Yes	1 (33.3)			0 (0.00)		
Personality Disorder						
No	119 (50.0)	5.17	0.02	82 (36.1)	14.12	0.00
Yes	14 (77.8)			14 (82.4)		
PTSD/OCD						
No	119 (50.0)	5.17	0.02	85 (37.4)	4.93	0.03
Yes	14 (77.8)			11 (64.7)		
ADHD						
No	123 (50.4)	4.97	0.03	90 (38.8)	0.60	0.44
Yes	10 (83.3)			6 (50.0)		
Other						
No	131 (52.0)	1.00	0.94	93 (38.8)	0.30*	0.14
Yes	2 (50.0)			3 (75.0)		
No history of Mental Health Diagnosis
	119 (61.0)	26.99	0.00	90 (48.6)	27.75	0.00
	14 (23.0)			6 (10.2)		
**On psychotropic medications**						
Antidepressants						
No	68 (44.4)	8.59	0.00	44 (30.8)	10.64	0.00
Yes	65 (63.1)			52 (51.5)		
Antipsychotics						
No	118 (49.8)	5.99	0.01	83 (36.7)	8.80	0.00
Yes	15 (78.9)			13 (72.2)		
Benzodiazepines						
No	123 (51.0)	1.38	0.24	87 (37.7)	5.14	0.02
Yes	10 (66.7)			9 (69.2)		
Mood stabilizers						
No	117 (50.2)	3.14	0.08	83 (37.2)	4.90	0.03
Yes	16 (69.6)			13 (61.9)		
Sleeping tablets						
No	111 (49.6)	4.13	0.04	80 (37.4)	2.81	0.09
Yes	22 (68.8)			16 (53.3)		
Stimulants for ADHD						
No	126 (50.6)	0.02*	0.01	94 (39.5)	1.00	0.76
Yes	7 (100)			2 (33.3)		
Other						
No	129 (52.2)	0.74*	0.65	91 (38.6)	0.27	0.17
Yes	4 (44.4)			5 (62.5)		
Not on any psychotropic medication						
No	83 (64.3)	15.99	0.00	62 (49.6)	11.30	0.00
Yes	50 (39.4)			34 (28.6)		
**Having received MH counselling in the past year**
No	52 (43.3)	6.73	0.01	32 (28.1)	11.40	0.00
Yes	81 (59.6)			64 (49.2)		
**Living in a region of AB/NS that has recently been impacted by the wildfires**
No	80 (51.0)	0.16	0.69	54 (36.2)	1.54	0.21
Yes	53 (53.5)			42 (44.2)		
**Having a wildfire evacuation order issued for the subscriber area of residence**
Yes	13 (54.2)	0.67*	0.60	12 (50.0)		
No	35 (51.5)			28 (43.8)	0.509	0.60
Not applicable	5 (71.4)			2 (28.6)		
**Having had to evacuate from your home due to the recent wildfires in AB/NS**
No	42 (53.8)	0.14	0.91	32 (42.7)	0.34	0.56
Yes	11 (52.4)			10 (50.0)		
**Have you lost any property because of the wildfire?**
No	51 (52.6)	1.77	0.18	40 (43.0)	0.19	0.11
Yes	2 (100.0)			2 (100.0)		
**Kind of property that was lost**						
Home	2 (100.0)	1.86	0.17	2 (100.0)	0.15	0.08
Car	133 (52.0)					
Farm	133 (52.0)					
**I lost no property**	51 (52.6)	0.02	0.88	40 (43.0)	0.85	0.36
**Having received support from family and friends in relation to the recent wildfire**
Absolute support	10 (40.0)	5.66	0.13	9 (36.0)	2.50	0.48
Some support	8 (44.4)			6 (35.3)		
Only limited support	12 (75.0)			8 (57.1)		
Not at all	23 (57.5)			19 (48.7)		
**Having received support from the government of AB/NS in relation to the recent wildfire**
Absolute support	0 (0.0)			0 (0.0)		0.27
Some support	5 (41.7)	0.19*	0.16	7 (58.3)	0.32	
Yes, but only limited support	5 (71.4)			4 (57.1)		
Not at all	43 (55.8)			31 (42.5)		
**Having received support from Red Cross in relation to the recent wildfire**
Yes some support	4 (80.0)	0.11	0.09	3 (60.0)	0.62	0.54
Only limited support	0 (0.0)			2 (66.7)		
Not at all	49 (53.8)			37 (42.5)		
**Frequency of watching television images about the devastation caused by the recent wildfires in AB/NS**
Daily	40 (44.4)			32 (37.6)		
About every other day	30 (61.2)	5.86	0.21	23 (46.9)		
About once a week	16 (64.0)			4 (18.2)	5.73	0.22
Less than once a week	15 (45.5)			13 (40.6)		
Haven’t watched TV images of the devastation	32 (54.2)			24 (42.9)		
**Having called the mental health criss line in relation to the recent wildfires in AB/NS**
No	131 (51.8)	1.00*	0.61	94 (38.8)	0.15	0.08
Yes	2 (66.7)			2 (100.0)		

*Fisher’s exact test.

Respondents who were ≤ 40 years old were more likely to present with low resilience (67.6%) and likely PTSD (55.1%) compared to other age groups. Also, unemployed respondents were more likely to present with low resilience (64.8%) and likely PTSD (53.7%) than those who were employed.

Furthermore, respondents with a history of common mental health conditions diagnosed by mental health professional were more likely to present with low resilience and likely PTSD symptoms compared with respondents who lack the respective health conditions. This included respondents diagnosed with depression (Low resilience: 63.3%; Likely PTSD: 51.4%), anxiety disorder (Low resilience: 66.2%; Likely PTSD: 49.6%), a history of personality disorder (Low resilience 77.8%; Likely PTSD: 82.4), and history of PTSD/OCD disorder (Low resilience: 77.8%; Likely PTSD:64.7%). Respondents with no previous mental health diagnosis were less likely to present with low resilience:23.0% and Likely PTSD:10.2%.

Regarding history of psychotropic medications, respondents using antidepressants (Low resilience:63.1%; Likely PTSD: 51.3%), Antipsychotics (Low resilience:78.9%; Likely PTSD: 72.2%) were more likely to develop low resilience and Likely PTSD symptoms, respectively, than respondents who were not on those medications. Likewise, respondents who were on benzodiazepines (69.2%), Mood stabilizers (61.9%), and other medications (28.6%) were more likely to present with probable PTSD symptoms compared to respondents who were not on the mentioned medications. Finally, the univariate analysis of the association between respondents’, low resilience, and the likelihood of PTSD shows that there was an association between the history of counselling and low resilience as well as the likelihood of presenting with PTSD symptoms.

Table 3 illustrates the multivariable binomial logistic regression model used to determine the potential predictors of low resilience symptoms among the study respondents. Overall, 14 variables had significant or near-significant association (*p* ≤ 0.1) with low resilience (Table 2). The logistic model was statistically significant; *Χ^2^* (df = 16; *n* = 256) = 60.1, *p* < 0.001, implying that the model could differentiate between respondents who had High to normal resilience and those with low resilience during the period of the recent wildfire in Alberta and Nova Scotia provinces. The model accounted for 20.9% (Cox and Snell *R*^2^) to 27.9% (Nagelkerke *R*^2^) of the variance. Inferring from the goodness-of-fit statistic using Hosmer-Lemeshow goodness-of-fit test, the model was adequately fit (Chi^2^ = 5.93; *p* = 0.66) and correctly classified 68.4% of cases.

**Table 3:** Logistic regression results of the significant association between study variables and likelihood to present with low resilience.

	B	S.E.	Wald	df	Sig.	Exp(B)	95% C.I. for EXP(B)	
							Lower	Upper
**Age**								
> = 60 yrs.			4.711	3	0.194			
50–59 yrs	−0.885	0.439	4.058	1	0.044	0.413	0.174	0.976
40–49 yrs	−0.372	0.406	0.841	1	0.359	0.689	0.311	1.527
<40 yrs	−0.649	0.432	2.261	1	0.133	0.522	0.224	1.218
**Employed**	−0.929	0.336	7.640	1	0.006	0.395	0.204	0.763
**History of depressive disorder**	−0.343	0.401	0.732	1	0.392	0.710	0.324	1.557
**History of anxiety disorder**	−0.708	0.373	3.609	1	0.057	0.493	0.237	1.023
**History of personality disorder**	0.332	0.720	0.212	1	0.645	1.394	0.340	5.715
**History of PTSD or OCD**	−0.495	0.651	0.580	1	0.446	0.609	0.170	2.180
**History of ADHD**	−1.020	0.874	1.360	1	0.244	0.361	0.065	2.002
**Received mental health diagnosis**	0.539	0.546	0.977	1	0.323	1.715	0.589	4.995
**On Antidepressants**	−0.065	0.341	0.037	1	0.848	0.937	0.481	1.826
**On Antipsychotics**	−0.914	0.751	1.482	1	0.223	0.401	0.092	1.747
**On Mood Stabilizers**	0.288	0.637	0.205	1	0.651	1.334	0.383	4.644
**On Sleeping Tablets**	−0.319	0.471	0.458	1	0.499	0.727	0.289	1.830
**ADHD medication**	−20.078	0.804	0.000	1	0.999	0.000	0.000	.
**Received mental health counselling in the past year**	0.027	0.312	0.007	1	0.932	1.027	0.557	1.894
**Constant**	23.251	0.84	0.000	1	0.999	1,252		

As indicated in Table 3, only one variable, employment status, independently predicted symptoms of low resilience in the model. Respondents who reported that they were unemployed were about two and half times more likely to experience low resilience compared to respondents who were employed (OR = 2.53; 95% CI: 1.31–4.89).

Table 4 illustrates the multivariable binomial logistic regression model used to determine the potential predictors of likely PTSD symptoms among the study respondents. Overall 20 variables had significant or near-significant association (*p* ≤ 0.1) (Table 2). However, the model included 19 of the 20 chi-squared predictor variables for the likely PTSD, after one variable*: The history of receiving other psychotropic medications* that showed a high correlation with other variables (r_s_ > +/−0.7) was excluded from the model. The logistic model was statistically significant; Χ2 (df = 25; *n* = 244) = 73.94, *p* < 0.001, implying that the model could differentiate between respondents who were least likely to have PTSD and those with the likelihood of having PTSD during the period of the recent wildfire in Alberta and Nova Scotia. The model accounted for 26.1% (Cox and Snell *R*^2^) to 35.4% (Nagelkerke *R*^2^) of the variance. Inferring from the goodness-of-fit statistic using Hosmer-Lemeshow goodness-of-fit test, the model was adequately fit (Chi2 = 13.21; *p* = 0.11) and correctly classified 76.2% of cases.

**Table 4:** Logistic regression results of the significant association between study variables and likelihood to present with likely PTSD.

	B	S.E.	Wald	df	Sig.	Exp(B)	95% C.I. for EXP(B)	
							Lower	Upper
**Age**								
> = 60			5.500	3	0.139			
50–59	−0.606	0.500	1.468	1	0.226	0.546	0.205	1.454
40–49	0.081	0.455	0.032	1	0.859	1.084	0.445	2.644
<40	−0.869	0.479	3.289	1	0.070	0.419	0.164	1.073
**Ethnicity**								
Caucasian			4.676	4	0.322			
Indegenous	−2.263	1.226	3.407	1	0.065	0.104	0.009	1.150
Asian	−2.030	1.360	2.226	1	0.136	0.131	0.009	1.890
Black/Hispanic	−2.154	1.525	1.996	1	0.158	0.116	0.006	2.304
Other	−1.091	1.603	0.463	1	0.496	0.336	0.015	7.771
**Currently in relationship**	−0.308	0.354	0.759	1	0.384	0.735	0.367	1.470
**Employed**	−1.017	0.369	7.621	1	0.006	0.362	0.176	0.744
**Housing status**								
Own home			2.001	2	0.368			
Rented accommodation	−0.816	0.577	1.998	1	0.158	0.442	0.143	1.371
Live with Family or Friends	−0.615	0.613	1.008	1	0.315	0.541	0.163	1.796
**History of depression**	−0.364	0.434	0.702	1	0.402	0.695	0.297	1.628
**History of bipolar Disorder**	−0.725	0.843	0.741	1	0.389	0.484	0.093	2.525
**History of anxiety**	0.164	0.393	0.174	1	0.676	1.179	0.545	2.548
**History of alcohol abuse**	−0.748	0.791	0.895	1	0.344	0.473	0.101	2.229
**History of drug abuse**	−0.036	0.875	0.002	1	0.967	0.965	0.174	5.365
**History of disorder**	−0.956	0.812	1.385	1	0.239	0.385	0.078	1.889
**History of PTSD or OCD**	−0.481	0.652	0.543	1	0.461	0.618	0.172	2.221
**Received mental health diagnosis**	1.404	0.672	4.358	1	0.037	4.071	1.090	15.207
**On antidepressants medication**	−0.286	0.358	0.636	1	0.425	0.751	0.372	1.517
**On antipsychotics medication**	−0.122	0.788	0.024	1	0.877	0.885	0.189	4.144
**On benzodiazepines**	−0.270	0.749	0.130	1	0.718	0.763	0.176	3.311
**On mood stabilizers**	0.245	0.730	0.112	1	0.738	1.277	0.305	5.341
**On sleeping tablets**	0.011	0.508	0.000	1	0.982	1.011	0.374	2.735
**Received mental health counselling in the past year**	−0.406	0.346	1.375	1	0.241	0.667	0.338	1.313
**Constant**	5.699	2.217	6.610	1	0.010	298.550		

As indicated in Table 4, two variables, employment status and receiving mental health diagnosis from a healthcare professional independently predicted symptoms of likely PTSD in the model. Respondents who responded that they were employed were 0.36 times less likely to present symptoms of likely PTSD than those who responded no to being employed (OR = 0.36; 95% CI: 0.18–0.74). Likewise, respondents who had a prior mental health diagnosis were about 4 times more likely to present symptoms of PTSD compared to respondents who had not received a mental health diagnosis (OR = 4.07; 95% CI: 1.09–15.21).

## Discussion

The overall prevalence of low resilience in our survey stood at 52.0%. This prevalence is higher than the 37.4% prevalence found among residents of Fort McMurray 5 years after the May 2016 wildfire (32) and 1 year post the 2020 flooding in Fort McMurray (37). The overall prevalence of likely PTSD in this study was 39.3%. This prevalence is comparable to the 39.6% reported by residents of Fort McMurray 5 years after the devastating wildfire outbreak (21). Conversely, the prevalence of likely PTSD in this study sample is higher than the 12.8 and 13.6% prevalence reported in the general population of Fort McMurray 6 months and 18 months after the 2016 wildfire, respectively (14, 26). It is important to re-state that data in this study was collected during the wildfires.

The mere uncertainty and unpredictability of a wildfire situation, including the rapidly changing conditions and the need for quick decisions might have led to elevated stress levels, which can contribute to the observed high prevalence of PTSD and low resilience symptoms compared with the prevalence reported in other studies following natural disasters. Consistent with this, the prevalence of any mental health condition was reduced from 20.6 to 10.9% according to the results from a study conducted 2 to 5 months post the Hurricane Ike natural disaster (38).

### Low resilience

Strengthening our ability to withstand disasters is highlighted as one of the United Nations’ 17 key objectives, for sustainable development by 2030. Specifically within the framework of goal 13 (climate action), there is an emphasis on improving our capacity to cope with disasters and climate-related risks (39). Therefore, building resilience in individuals plays a role, in ensuring that disaster management efforts are effective, efficient, and comprehensive. Resilient individuals are characterized by their sustainability, self-sufficiency, satisfaction, and prosperity.

The results from our study indicate that one variable, unemployment significantly predicted low resilience among respondents when all other variables are controlled for in the regression model. Unemployed respondents were about 3 times more likely to present with low resilience compared to those who were employed. The association between unemployment and negative mental health outcomes has been extensively explored and documented in the literature (40–43). Unemployment is associated with lower psychosocial well-being, decreased life satisfaction, and an elevated likelihood of developing poor mental health due to its impact on individuals (42). Conversely, our result is inconsistent with a study conducted 5 years after the wildfire in Fort McMurray which assessed the prevalence and predictors of low resilience among the respondents. Their results indicated that unemployment was not significantly associated with low resilience when all the factors in the model were controlled for (32). It is important to take into account the impact of trauma, on aspects of adaptation. However, more research is needed to understand how unemployment and low resilience are related among individuals affected by disasters both during and, after the event.

Our results further suggest that age was not a significant predictor of low resilience. This is inconsistent with a study conducted 5 years after the wildfire in Fort McMurray which reported that age was significantly correlated with low resilience. In addition, the study by Yu et al. (44) reported that younger age students had a greater level of resilience than older students while other studies suggested a negative correlation between resilience and age (44, 45).

### Post traumatic stress disorder

Regarding PTSD, the results suggested that two variables, unemployment and having no history of any mental health diagnosis from a professional predicted the likelihood of PTSD in respondents. Participants who responded that they were employed were less likely to experience PTSD (OR = 0.36; 95% CI: 0.18–0.74). That is unemployed respondents were about 2.8 times more likely to present with symptoms of likely PTSD when compared with those who were employed. Our result is consistent with a study conducted 5 years after the devastating wildfires in Fort McMurray in which their findings suggested that unemployed respondents were more likely to present with probable PTSD (100%) when compared with respondents who were employed (21). Again, our result is consistent with a previous study conducted 1 year after the September 11 attacks. The study reported that unemployment predicted the likelihood of PTSD in the entire cohort (*p* = 0.02) of study participants (46). To confirm a causal relationship between employment status and PTSD, more detailed information would have to be sourced from future studies. Nevertheless, our result suggests a specific relationship between PTSD and employment.

Furthermore, our results indicated that respondents who reported having a history of mental health diagnosis were more likely to present with symptoms of PTSD when compared to respondents who had not received a mental health diagnosis. This finding is consistent with previous studies that evaluated the predictors of likely PTSD. Findings from previous wildfire-impacted respondents suggested that respondents who reported having a history of depression before the wildfire were more likely to report PTSD after the wildfire (21). Similarly, in a related post-wildfire study 6 months after the Fort McMurray wildfires, participants with a history of anxiety disorder before the wildfire were more likely to report PTSD symptoms compared to those without a prior history of Anxiety disorder (14).

To gain an understanding of the predictors of likely PTSD post-wildfire disasters, more research should focus on studies that delve into the various factors that contribute to discrepancies in PTSD symptoms between individuals with and without a history of any mental health diagnosis.

### Limitations

This study, like most studies that assess and evaluate post-disaster conditions, presents with limitations. First, this study adopted a cross-sectional survey design which limits the development of the causal relationship between variables of interest and both low resilience and PTSD. Second, the sample size for the study was smaller than anticipated, which may impact the generalizability of the study findings. Again, the results of our study are from a random sample of Text4Hope subscribers with a non response rate of about 84 and 81% for AB and NS, respectively. Hence the findings do not generalize to the population of Alberta and Nova Scotia. In addition, given that the sample size of 298 for this study was much lower than the predicted sample size of 385, the actual margin of error for our prevalence estimates for likely PTSD was 5.79%, which was higher than the projected 5% determined apriori. Finally, an important limitation pertains to the continued utilization of the DSM-IV PCL-C, despite the current iteration being the DSM-5 for reasons of consistency.

### Study implications

This study offers crucial clinical implications for mental health interventions in the context of wildfires. The integration of Text4Hope showcases the importance of incorporating digital tools for data collection and mental health support during wildfires. Unemployment emerges as a significant predictor of both low resilience and likely PTSD, urging clinicians to adopt inclusive strategies that address diverse socioeconomic factors in disaster-affected populations. Additionally, the association between a history of mental health diagnosis and likely PTSD underscores the need for personalized psychosocial support, guiding the development of targeted interventions for individuals with pre-existing mental health conditions. Overall, this research provides valuable insights for clinicians, policymakers, and public health experts, emphasizing the potential of technology, the necessity of inclusivity, and the importance of personalized approaches in enhancing mental health outcomes during and after disasters.

## Conclusion

Natural disasters will continue to affect humans and their communities, and all efforts should be made to minimize their impact on the overall mental health of affected individuals. This study aimed to identify the predictors of low resilience and likely PTSD symptoms among our study respondents; these predictors include unemployment and a history of mental health diagnosis. This study provides valuable clinical interventional directions for the development of appropriate psychosocial support programs for at-risk populations during and post devastating natural disasters like wildfires.

## Data availability statement

The raw data supporting the conclusions of this article will be made available by the authors, without undue reservation.

## Ethics statement

The studies involving humans were approved by the study received ethical approval from the Health Research Ethics Board of the University of Alberta (Pro00086163) and the Research Ethics Board at Nova Scotia Health (REB file #1028254). The studies were conducted in accordance with the local legislation and institutional requirements. The participants provided their written informed consent to participate in this study.

## Author contributions

MA: Data curation, Formal analysis, Methodology, Writing – original draft, Writing – review & editing. RS: Data curation, Formal analysis, Methodology, Writing – review & editing. BA: Data curation, Project administration, Writing – review & editing. RD: Data curation, Methodology, Project administration, Writing – review & editing. VA: Conceptualization, Data curation, Formal analysis, Funding acquisition, Investigation, Methodology, Project administration, Resources, Supervision, Validation, Visualization, Writing – review & editing.
